# Electronegative very-low-density lipoprotein induces brain inflammation and cognitive dysfunction in mice

**DOI:** 10.1038/s41598-021-85502-0

**Published:** 2021-03-16

**Authors:** Ying-Shao Lin, Ching-Kuan Liu, Hsiang-Chun Lee, Mei-Chuan Chou, Liang-Yin Ke, Chu-Huang Chen, Shiou-Lan Chen

**Affiliations:** 1 Graduate Institute of Medicine, College of Medicine, Kaohsiung Medical University (KMU), 100 Shiquan 1st Rd, Sanmin Dist., Kaohsiung City, 807 Taiwan; 2 Department of Neurology, KMU Hospital, KMU, Kaohsiung, Taiwan; 3 Department of Neurology, Faculty of Medicine, College of Medicine, KMU, Kaohsiung, Taiwan; 4 Division of Cardiology, Department of Internal Medicine, KMU Hospital and Faculty of Medicine, College of Medicine, KMU, Kaohsiung, Taiwan; 5 Lipid Science and Aging Research Center, College of Medicine, KMU, Kaohsiung, Taiwan; 6 Graduate Institute of Clinical Medicine, College of Medicine, KMU, Kaohsiung, Taiwan; 7 Department of Neurology, Kaohsiung Municipal Ta-Tung Hospital, KMU, Kaohsiung, Taiwan; 8 Department of Medical Laboratory Science and Biotechnology, KMU, Kaohsiung, Taiwan; 9 Vascular and Medicinal Research, Texas Heart Institute, Houston, TX USA; 10 Department of Medical Research, KMU Hospital, Drug Development and Value Creation Research Center and MSc Program in Tropical Medicine, KMU, Kaohsiung, Taiwan

**Keywords:** Cognitive neuroscience, Neuroimmunology

## Abstract

Epidemiologic studies have indicated that dyslipidemia may facilitate the progression of cognitive dysfunction. We previously showed that patients with metabolic syndrome (MetS) had significantly higher plasma levels of electronegative very-low-density lipoprotein (VLDL) than did healthy controls. However, the effects of electronegative-VLDL on the brain and cognitive function remain unclear. In this study, VLDL isolated from healthy volunteers (nVLDL) or patients with MetS (metVLDL) was administered to mice by means of tail vein injection. Cognitive function was assessed by using the Y maze test, and plasma and brain tissues were analyzed. We found that mice injected with metVLDL but not nVLDL exhibited significant hippocampus CA3 neuronal cell loss and cognitive dysfunction. In mice injected with nVLDL, we observed mild glial cell activation in the medial prefrontal cortex (mPFC) and hippocampus CA3. However, in mice injected with metVLDL, plasma and brain TNF-α and Aβ-42 levels and glial cell activation in the mPFC and whole hippocampus were higher than those in control mice. In conclusion, long-term exposure to metVLDL induced levels of TNF-α, Aβ-42, and glial cells in the brain, contributing to the progression of cognitive dysfunction. Our findings suggest that electronegative-VLDL levels may represent a new therapeutic target for cognitive dysfunction.

## Introduction

As the world’s older population is growing at a rapid rate, the number of patients with neurodegeneration is rising. Identifying novel pathogenic factors that lead to neurodegeneration and developing strategies to prevent it have become primary clinical and public goals. Alzheimer's disease (AD) and non-AD cognitive dysfunction are examples of neurodegenerative diseases that share similar features, including the chronic progression of cognitive dysfunction, memory loss, continual neuronal apoptosis, and brain atrophy. Previously, several factors such as neurotransmitter deficiency^1, 2^, oxidative stress^3^, and neuronal inflammation^4^ have been identified as therapeutic targets to delay or prevent the onset and progression of neurodegenerative diseases. Those targets have formed the basis of current treatment modalities, which mostly mitigate disease symptoms. However, in addition to diminishing the symptoms of neurodegeneration, halting the progression of neurodegeneration is essential for improving functionality and quality of life. Therefore, identifying novel risk factors that play a role in the mechanism of neurodegeneration is of urgent clinical importance.

Dyslipidemia and metabolic syndrome (MetS) are considered chronic risk factors for neurodegeneration, reasonably because of their pathogenic roles in cardiovascular diseases. An epidemiologic study showed that AD is significantly more likely to develop in elderly patients with MetS than in those without MetS (7.2% vs. 2.8%, p < 0.001)^5^. Furthermore, in a 20-year follow-up study, middle-aged people with higher blood lipid levels presented with greater cognitive decline than did those with healthy lipid profiles^6^. Others have reported that long-term high-fat diets induce cognitive dysfunction in rodents^7–11^. However, the roles of blood lipid molecules in the central nervous system (CNS) and cognitive function are not well understood.

In elderly patients with hyperlipidemia, cognitive dysfunction does not always occur^5^, indicating that more precise research is needed to understand the relationship between blood lipids and cognitive dysfunction. Several studies have shown that very-low-density lipoprotein (VLDL)—a major lipoprotein in the blood—is important for developing dyslipidemia. The overproduction of VLDL is the hallmark of dyslipidemia in patients with MetS^12^. In previous studies, we showed using anion-exchange chromatography that VLDL can be subclassified according to electrical properties into different charge grades on the basis of its degree of surface anodization^13^. We found that the most electronegative VLDL (ie, V5) but not the least electronegative VLDL (ie, V1) can induce endothelial cell apoptosis^13^. Patients with MetS had a significantly higher percentage of V5 (MetS, 47.9% vs. healthy control, 34%) and plasma V5 concentration (MetS, 15.2 mg/dL vs. healthy control, 5.5 mg/dL) than did healthy controls^13^. Moreover, the chronic exposure of young adult mice to VLDL from patients with MetS (metVLDL) induced cardiomyocyte apoptosis and left atrial enlargement, providing new insight into cardiovascular disease induced by VLDL^14^. These findings suggest that the elevated levels of electronegative VLDL in patients with MetS play a role in damaging cardiomyocytes, resulting in compromised heart function. Therefore, we believe that VLDL's cardiocytotoxicity is not determined by its absolute concentration in the plasma but by its electronegativity.

To better understand the relationship between VLDL electronegativity and cognitive dysfunction, we studied the pathologic effects of electronegative VLDL on cognitive function and the CNS. By injecting VLDL preparations with different degrees of electronegativity into the tail vein of mice, we were able to compare their long-term effects on the brain and behavior. We hypothesized that long-term exposure to VLDL with increased electronegativity, such as in patients with MetS, may deteriorate neuronal function.

## Results

### Cognitive function in mice

**Figure 1:**
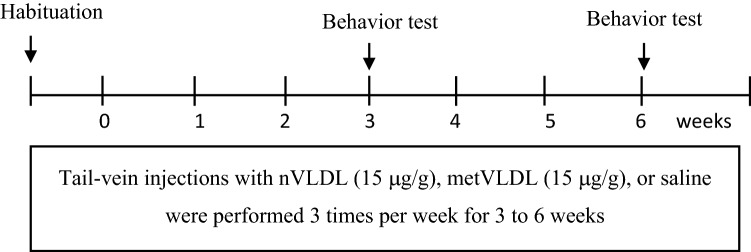
Schematic diagram showing the experimental protocol used for VLDL tail-injection studies in mice.

**Figure 2:**
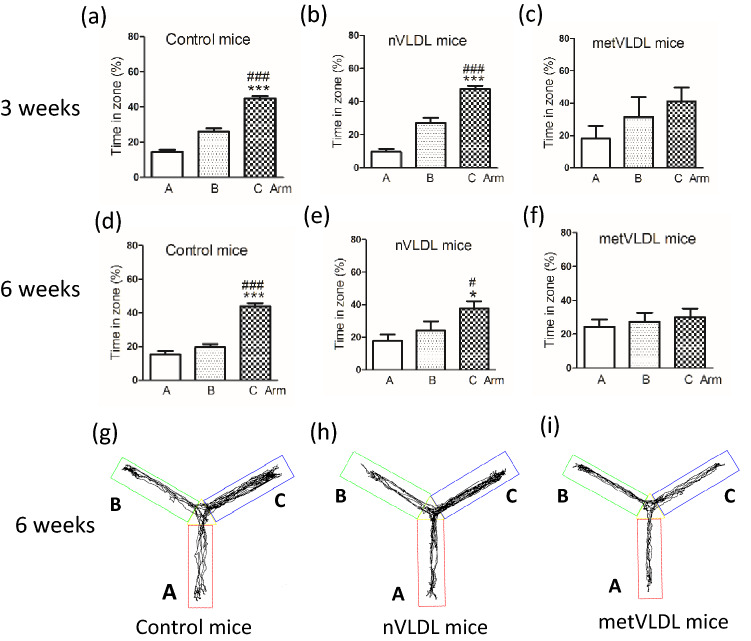
Y maze cognitive function test in mice. B6 mice were injected in the tail vein 3 times per week for 3–6 consecutive weeks with nVLDL (15 μg/g), metVLDL (15 μg/g), or an equivalent volume of saline (control). The percentage (%) of time spent in the start arm A, familiar arm B, or novel arm C after 3 weeks (**a**–**c**) or 6 weeks (**d**–**f**) of injections is shown for the indicated group. The moving track of control (**g**), nVLDL (**h**), and metVLDL (**i**) mice is shown, determined by using video-tracking software (Panlab Smart video-tracking software, version 3.0). Data are presented as the mean ± standard error of the mean (n ≥ 12 in each group). *p < 0.05, ***p < 0.0001 arm C versus A, #p < 0.05, ###p < 0.0001 arm C versus B within each group. P-values were determined by using one-way analysis of variance with the Newman-Keuls post-hoc test.

Using the Y maze behavior test, we examined cognitive function in mice that were injected in the tail vein with saline (control), VLDL from healthy volunteers (nVLDL), or metVLDL 3 times a day for 3–6 weeks (Fig. 1). After 3 weeks of injections, saline-treated and nVLDL-treated mice spent significantly more time in the novel arm C than in the familiar arm B (control: arm C vs. B, p < 0.0001, Fig. 2a; nVLDL: arm C vs. B, p < 0.0001, Fig. 2b), indicating normal recognition of a new space in saline-treated and nVLDL-treated mice. However, in metVLDL-treated mice, we observed dysfunctional arm C and B recognition (arm C vs. B, 41.0% vs. 31.6%, p = 0.54; Fig. 2c). After an additional 3 weeks of injections (6 weeks total), dysfunctional arm C and B recognition persisted in metVLDL-treated mice (arm C vs. B, p = 0.25; Fig. 2f,i), which was not observed in control (arm C vs. B, p < 0.0001; Fig. 2d,g) and nVLDL-treated mice (arm C vs. B, p = 0.02; Fig. 2e,h).

### Plasma Aβ-42 and TNF-α expression in mice

We also examined the plasma and brain tissues of mice after 6 weeks of injections. Plasma Aβ-42 levels were markedly higher in metVLDL-treated mice (but not nVLDL-treated mice) than in saline-treated mice (p = 0.06, Table 1). In addition, plasma TNF-α levels were significantly higher in metVLDL-treated mice (but not nVLDL-treated mice) than in saline-treated mice (p = 0.05, Table 1).

**Table 1:** Protein expression analysis in mouse plasma and brain after 6 weeks of tail-vein injections with nVLDL, metVLDL, or saline.

Variable	Saline (control)	nVLDL	metVLDL	*F* value	*P* value
	n = 12	n = 12	n = 13		
**Plasma**					
Aβ42 (pg/mL)	80.0 ± 28.4	61.7 ± 26.3	202.5 ± 47.5	2.6	0.06
TNF-α (pg/mL)	0.5 ± 0.5	1.6 ± 1.0	4.0 ± 1.2*	3.9	0.05
**Brain tissue**					
Cortex Aβ42 (pg/mg tissue)	55.5 ± 4.1	77.2 ± 7.1*	84.7 ± 7.2*	5.6	0.01
mPFC TNF-α (pg/mg protein)	10.4 ± 1.3	13.3 ± 1.5	17.9 ± 2.8*	3.6	0.04
Hip TNF-α (pg/mg protein)	7.6 ± 1.6	12.4 ± 2.8	30.8 ± 10.9	3.0	0.07

Aβ42, amyloid beta 42; mPFC, medial prefrontal cortex; Hip, hippocampus; nVLDL, VLDL from healthy volunteers; metVLDL, VLDL from patients with metabolic syndrome. Data are presented as the mean ± standard error of the mean.

*p < 0.05 versus control group. P-values were determined by using one-way analysis of variance with the Newman-Keuls post-hoc test.

### Brain Aβ-42 and TNF-α expression in mice

After 6 weeks of injections, cortex levels of Aβ-42 in nVLDL-treated mice and metVLDL-treated mice were significantly higher than those in saline-treated mice (p < 0.05 for both, Table 1). In addition, medial prefrontal cortex (mPFC) levels of TNF-α were significantly higher in metVLDL-treated mice (but not nVLDL-treated mice) than in saline-treated mice (p < 0.05, Table 1).

### Brain microglia in mice

**Figure 3:**
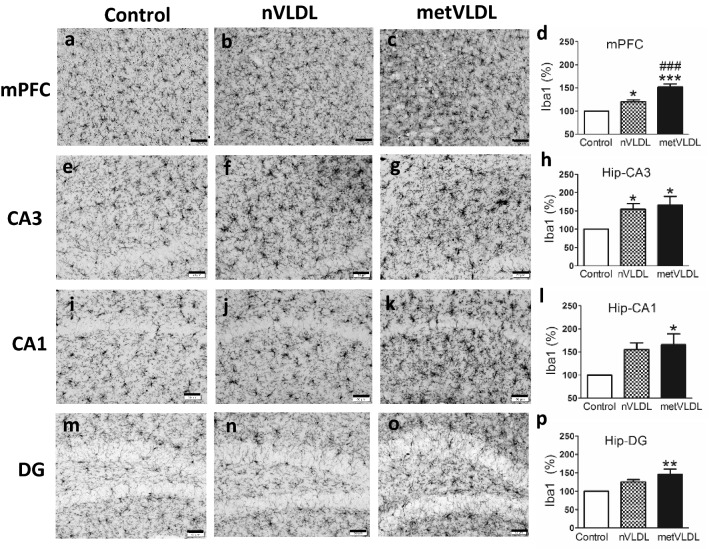
Immunostaining showing the expression of Iba1 (microglia) in the medial prefrontal cortex (mPFC) and hippocampus CA3, CA1, and dentate gyrus (DG) of mice after 6 weeks of tail-vein injections with saline (control: **a**, **e**, **i**, **m**), 15 μg/g nVLDL (**b**, **f**, **j**, **n**), or 15 μg/g metVLDL (**c**, **g**, **k**, **o**). Magnification = 20X; scale bar = 50 μm. The percentage of Iba1 immunostaining (%) in mPFC (**d**), CA3 (**h**), CA1 (**l**), and DG (**p**), calculated by using cellSens Dimension software (version 1.13, Olympus), is compared among groups (n ≥ 7 in each group). Data are presented as the mean ± standard error of the mean. *p < 0.05, **p < 0.01, ***p < 0.001 vs. control. ###p < 0.001, nVLDL vs. metVLDL. P-values were determined by using one-way analysis of variance with the Newman-Keuls post-hoc test.

After 6 weeks of injections, immunostaining for the microglia marker Iba1 in the mPFC was significantly higher in nVLDL-treated (p < 0.05, Fig. 3b,d) and metVLDL-treated mice (p < 0.001, Fig. 3c,d) than in saline-treated mice (Fig. 3a,d). The expression of Iba1 in the mPFC of metVLDL-treated mice was significantly higher than that of nVLDL-treated mice (metVLDL vs. nVLDL, p < 0.0001; Fig. 3d).

Immunostaining for the microglia marker Iba1 in the hippocampus cornu ammonis area 3 (CA3) was significantly higher in nVLDL-treated mice (p < 0.05, Fig. 3f,h) than in saline-treated mice (Fig. 3e,h), whereas it was not in CA1 (Fig. 3j,l) or the dendate gyrus (DG; Fig. 3n,p). However, in metVLDL-treated mice, immunostaining for the microglia marker Iba1 in hippocampus CA3 (p < 0.05; Fig. 3g,h), CA1 (p < 0.05, Fig. 3k,l), and DG (p < 0.01, Fig. 3o,p) was significantly higher than that in saline-treated mice (Fig. 3e,i,m), suggesting that nVLDL and metVLDL can prime microglial brain cells to different degrees.

### Brain astrocytes in mice

**Figure 4:**
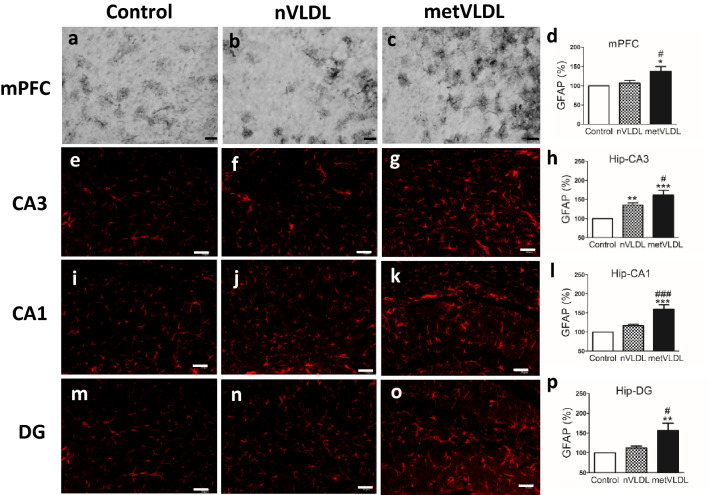
Immunostaining showing the expression of GFAP (astrocytes) in the medial prefrontal cortex (mPFC) and hippocampus CA3, CA1, and dentate gyrus (DG) of mice after 6 weeks of injections with saline (control: **a**, **e**, **i**, **m**), 15 μg/g nVLDL (**b**, **f**, **j**, **n**), or 15 μg/g metVLDL (**c**, **g**, **k**, **o**). Magnification = 20X; scale bar = 50 μm. The percentage of GFAP immunostaining (%) in mPFC (**d**), CA3 (**h**), CA1 (**l**) and DG (**p**), calculated by using cellSens Dimension software (version 1.13, Olympus), is compared among groups (n ≥ 7 in each group). Data are shown as the mean ± standard error of the mean. *p < 0.05, **p < 0.01, ***p < 0.001 vs. control. #p < 0.05, ###p < 0.001, nVLDL vs. metVLDL groups. P-values were determined by using one-way analysis of variance with the Newman-Keuls post-hoc test.

After 6 weeks of injections, immunostaining for the astrocyte marker anti-glial fibrillary acidic protein (GFAP) in the mPFC was significantly higher in metVLDL-treated (p < 0.05, Fig. 4c,d) but not nVLDL-treated mice (Fig. 4b,d) than in saline-treated mice (Fig. 4a,d). Immunostaining for the astrocyte marker GFAP in hippocampus CA3 (p < 0.01, Fig. 4f,h) was significantly higher in nVLDL-treated mice than in saline-treated mice, whereas it was not in CA1 (Fig. 4j,l) or DG (Fig. 4n,p). Immunostaining for GFAP in hippocampus CA3 (p < 0.0001, Fig. 4g,h), CA1 (p < 0.0001, Fig. 4k,l), and DG (p < 0.01, Fig. 4o,p) was significantly higher in metVLDL-treated mice than in saline-treated mice (Fig. 4e,i,m). Moreover, immunostaining for the astrocyte marker GFAP in the mPFC and hippocampus was significantly higher in metVLDL-treated mice than in nVLDL-treated mice (metVLDL vs. nVLDL, p < 0.05; Fig. 4d,h,l,p). These data suggest that nVLDL and metVLDL can increase the population of brain astrocytes to different degrees.

### Brain neuronal cells in mice

**Figure 5:**
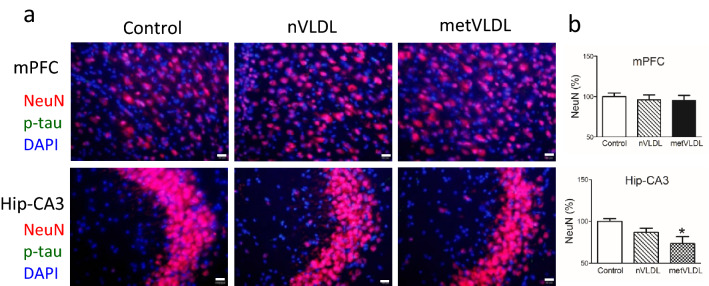
Immunofluorescence staining showing the expression of neuronal cells (NeuN, red) and phosphorylated tau (p-tau, green) in the (**a**) medial prefrontal cortex (mPFC) and hippocampus CA3 (Hip-CA3) of mice after 6 weeks of injections with saline (control), 15 μg/g nVLDL, or 15 μg/g metVLDL. Magnification = 40X; scale bar = 20 μm. (**b**) The percentage of NeuN immunostaining (%) in the mPFC and hippocampus CA3, calculated by using cellSens Dimension software (version 1.13, Olympus), is compared among groups (n ≥ 4 in each group). Data are shown as the mean ± standard error of the mean. *p < 0.05 vs. control. P-values were determined by using one-way analysis of variance with the Newman-Keuls post-hoc test.

In immunostaining studies, expression of the neuronal cell marker NeuN in the hippocampus CA3 was significantly lower in metVLDL-treated mice than in saline-treated mice (p < 0.05, Fig. 5). In the mPFC, expression of the NeuN marker was similar among nVLDL-treated, metVLDL-treated, and control mice (Fig. 5). Furthermore, the downregulation of CA3 neuronal cells in the hippocampus of mice treated with metVLDL was associated with high levels of TNF-α expression (Fig. S1).

In addition, we used immunostaining to examine the expression of phosphorylated tau protein in the mPFC and hippocampus and found no significant difference in the expression of phosphorylated tau in the brain tissue of nVLDL-treated and metVLDL-treated mice (Fig. 5a).

### Brain VLDL receptor in mice

We examined the expression of VLDL receptor (VLDLR) in the brain of mice after 6 weeks of VLDL injections. The expression of VLDLR and molecules involved in its signaling transduction pathway such as phosphorylated PI3 kinase p85 (phospho-PI3K) was no different in mice treated with nVLDL or metVLDL than in control mice (Figure S2).

## Discussion

Our study findings provide new insight into the relationship between blood lipids and cognitive function. We showed that the long-term (ie, 6-week) tail-vein injection of metVLDL but not nVLDL resulted in significantly increased cognitive dysfunction in mice when compared with control mice. Furthermore, metVLDL induced higher levels of TNF-α in the plasma and brain, Aβ-42 in the brain, and glial cell expression than did nVLDL. Although the injection of human VLDL may have induced a minor immunologic reaction in our mouse model, our data show direct in vivo evidence that nVLDL and metVLDL exert different effects on cognitive function, glial activation, and Aβ-42 and cytokine expression in the brain. To our knowledge, this study is the first to show how VLDL electronegativity affects the CNS.

In the blood, VLDL is the primary carrier of triglycerides. VLDL is composed of approximately 35% cholesterol, 35% phospholipids, and various apolipoproteins; it facilitates lipoprotein formation and function^15^. Several studies that have investigated the effects of abnormal VLDL on cognitive function have focused on the role of dysfunctional apolipoproteins in VLDL. For example, apolipoprotein E (ApoE) in VLDL binds to VLDL/LDL receptors, allowing hepatocytes to consume lipoproteins and metabolize cholesterol to regulate blood lipid levels^16^. In patients with an E4/E4 *ApoE* genotype, the risk of developing AD is increased by 5 to 15 times^17^. In these patients, poor metabolism or transportation of ApoE by VLDL particles was presumed to be a possible risk factor for developing AD.

Although the elevation of blood lipids has been shown to negatively affect the brain, cognitive dysfunction did not occur in all patients with hyperlipidemia^5^. Furthermore, cognitive dysfunction developed in only 7.2% of elderly patients with hyperlipidemia^5^. As we reported previously, the concentration of electronegative VLDL (V5) was 15.2 mg/dL in metVLDL and 5.5 mg/dL in nVLDL^13^. In the present study, we showed that injections with metVLDL or nVLDL produced different degrees of inflammatory responses and cognitive dysfunction in mice. These data provide new insight into the role of VLDL in maintaining cognitive function.

In the brain, VLDLR is an ApoE receptor that is expressed on the endothelium of capillaries and small arterioles^18^, as well as on resting and activated microglia^19^. The overactivation of VLDLR may induce the phosphorylation of Dab1, which leads to activation of PI3K/Akt^20^ and the phosphorylation of tau protein^21^. In this study, we observed no significant difference in the expression of VLDLR or its signaling molecules in the mouse brain after chronic administration of nVLDL or metVLDL. However, we found that plasma and brain TNF-α levels were significantly higher in metVLDL-treated mice than in saline-treated mice. Given that VLDL is involved in the inflammatory response of adipose tissue^22^ and the modulation of cytokine secretion from lymphocytes^23^, we speculate that metVLDL may induce a greater inflammatory response in the peripheral blood system than nVLDL. TNF-α in the peripheral blood has been shown to pass through the blood–brain barrier and induce neuronal inflammation^24^. Indeed, Qin et al.^24^ showed that the systemic administration of lipopolysaccharide did not reach the brain; however, within hours, it induced early TNF-α expression in the serum and liver, as well as long-lasting TNF-α expression in the brain for days, with subsequent microglial activation and neuronal damage. Thus, peripheral inflammation may initiate or contribute to the neuroinflammation and neurodegeneration in the CNS^24^. In this study, we found that CA3 neurons were significantly downregulated in mice injected with metVLDL and that TNF-α expression was higher in the hippocampus of these mice (Table 1), indicating a possible mechanism of metVLDL-induced circulatory cytokine expression, neuronal inflammation, and neuronal damage. Thus, the systemic inflammation induced by metVLDL may explain the glial cell activation and neuronal loss in the hippocampus of metVLDL-treated mice that we observed, which may in turn lead to the subsequent development of cognitive dysfunction.

We recently showed in primary mouse microglia and mixed glial cell cultures that the single-dose administration of metVLDL but not nVLDL activated microglial cells and increased levels of TNF-α^25^. In the present study, the repeated administration of nVLDL or metVLDL for a long-term duration (ie, 6 weeks) resulted in different degrees of microglial activation in the brain's cognition-related areas. Mice treated with metVLDL had higher levels of TNF-α and glial-cell expression in the brain than did mice treated with nVLDL, revealing the ability of electronegative VLDL to induce cytokine expression and neuronal inflammation in vitro^25^ and in vivo. In the CNS, glial cells are activated in response to biologic insults such as pathogen invasion^26^ and a dyslipidemic microenvironment^27^. In mouse models of neuronal degeneration, activated brain microglia have been shown to play a role in neuron dysfunction^28–31^. Microglia are also involved in obesity-associated cognitive decline in mice^29^. Moreover, activated brain microglia mediate early synapse loss in mouse models of AD^32^, whereas eliminating the microglia in mice with AD prevents neuronal loss^33^. Thus, the activation of microglial cells by systemic electronegative VLDL may subsequently lead to the decline of cognitive function.

After mice were injected with VLDL for 6 weeks, we observed an increase in astrocytes in the brain, as shown by GFAP staining, especially in metVLDL-treated mice. Previous studies have shown that astrocytes are activated along with microglial cells in the traumatically injured mouse brain^34^ and that this progresses into degenerative neuronal disease^35^. Increased GFAP expression has been considered as a hallmark of astrocyte activation in neurodegenerative disorders^35^. Our data support a relationship between electronegative VLDL and the pathogenesis of cognitive dysfunction and suggest that nVLDL and metVLDL affect brain microglia and astrocytes to different degrees.

We also observed a systemic increase in Aβ-42 in metVLDL-treated mice after 6 weeks. Furthermore, the expression of Aβ-42 was significantly increased in the brain of the metVLDL group. These findings suggest that a higher percentage of electronegative VLDL induces systemic and brain inflammation to affect amyloid-beta metabolism. Aβ-42 oligomers have been proposed as a basis for cognitive decline in AD^36^. In a transgenic mouse model of AD, diet-induced hyperlipidemia resulted in the accumulation of Aβ42^37^. Further study showed that activated brain microglia are involved in the accumulation of Aβ plaques^37^. Therefore, we believe that electronegative VLDL induces systemic and brain inflammation, which may subsequently affect the production of Aβ-42 to cause neuronal dysfunction.

## Conclusions

Our findings support that higher levels of electronegative VLDL in the blood induce systemic inflammation, neuronal inflammation, and Aβ-42 accumulation in the brain, resulting in adverse effects on cognitive function. Thus, monitoring and managing the levels of electronegative VLDL may represent a new adjunct therapeutic modality for patients with degenerative neuronal diseases.

This study has some limitations. First, although we injected mice with VLDL for 6 weeks, an even longer experimental duration should be considered in future studies to more closely match the conditions of patients with chronic dyslipidemia. Second, we did not examine the dynamic changes of VLDL in the brain of mice after the long-term administration of VLDL. Third, although we did not observe significant changes in the expression of VLDLR in the brain of mice 6 weeks after VLDL injection, the interaction between VLDL and VLDLR remains to be clarified. Labeling VLDL with lipophilic fluorescent dye as we have done previously with LDL^38^ may be useful for performing additional studies in vitro and in vivo.

## Methods

### Animal studies

All animal experiments were approved by the Institutional Animal Care and Use Committee (IACUC) of Kaohsiung Medical University (KMU) and were performed in accordance with the Association for Assessment and Accreditation of Laboratory Animal Care International (AAALAC) regulations, the US Department of Agriculture Animal Welfare Act, and the National Institutes of Health (NIH) Guide for the Care and Use of Laboratory Animals.

Nine-month-old male B6 mice were purchased from the National Laboratory Animal Center in Taiwan and were kept in an environmentally controlled room in an AAALAC-certified breeding facility of KMU (temperature: 23 ± 2 °C; 12-h/12-h light/dark cycle with light on from 0700 to 1900). Mice had free access to food and water. All experimental procedures adhered to the ethical standards established by the IACUC ethics committee and the guidelines of ARRIVE (Animal Research: Reporting of In Vivo Experiments).

### Isolation of human VLDL

VLDL was isolated from the serum of human blood samples by using sequential ultracentrifugation, as previously described^14^. Briefly, nVLDL and metVLDL were isolated from healthy volunteers (2 men and 2 women; mean age, 36 ± 8 years) and individuals who met the criteria for MetS according to the National Cholesterol Education Program–Adult Treatment Panel III guidelines (5 men, mean age, 48 ± 5 years), respectively^39^. All participants gave informed consent. The study was performed in accordance with Helsinki Declaration principles and was approved by the KMU Hospital Ethics Review Board (approved protocol number, KMUH-IRB-20130351). Total VLDL (density = 0.930–1.006 g/mL) was isolated by using sequential ultracentrifugation^13^. The protein concentration of VLDL was determined by using the bicinchoninic acid method.

### Y maze behavior test

We quantified cognitive function in mice by using the Y maze test, which is a behavioral test used to measure the tendency of rodents to explore new environments. Rodents with normal cognitive function typically prefer to investigate a new arm of the maze rather than return to one previously visited^40^. The cognition and short-term memory–related areas of the brain such as the mPFC and hippocampus are involved in this task. Testing is performed in a maze with 3 white plastic arms: A, the start arm; B, the familiar arm; and C, the novel arm. The dimensions of each arm are as follows: 30 cm (length) × 8 cm (width) × 15 cm (height). These arms were placed at 120° angles to form a Y-shape with a medial area in the center.

Three times a week for 6 consecutive weeks, the experimental mice were injected in the tail vein with nVLDL (15 μg/g) or metVLDL (15 μg/g), and the control mice were injected with equivalent volumes of saline. After 3 or 6 weeks of injection, the mice underwent behavioral tests (Fig. 1). At the beginning of each test day, the mice were habituated to the testing room for 60 min. Room brightness was kept consistent, and there were no disturbing sounds or odors during the tests. The same experimenter conducted all tests. A camera and an authorized image-tracking software (Panlab Smart video-tracking software, version 3.0) were used to monitor and analyze mouse behavior.

The test consisted of a 5-min sample trial (T1) and a 5-min retrieval trial (T2). In T1, the mouse was placed at the end of arm A and allowed to freely explore arm A and B, with arm C blocked. After the sample trial, the mouse was returned to its home cage for a 60-min intertrial interval. In T2, the mouse was placed into the medial area and then allowed to access all three arms of the maze. If a mouse climbed on the maze wall, it was immediately returned to the maze arm that it had abandoned. After each test and between T1 and T2, the maze was wiped with a 75% alcohol solution to prevent odor cues. The number of arm entries, the travel distance, and the time spent in each area were recorded and analyzed.

### Detection and quantification of plasma and brain TNF-α

After the behavioral tests, the mice were euthanized with an overdose of pentobarbital. Blood was collected via cardiac puncture and was centrifuged at 3000 × *g* at 4 °C for 10 min. The plasma fraction was isolated and immediately stored at –80 °C. The brain was dissected and cut into two halves. The left hemi-brain was separated into the areas related to cognitive, learning, and memory functions such as the cortex, mPFC, and hippocampus. The mPFC and hippocampus were then homogenized with phosphate-buffered saline (PBS) supplemented with 1X protease inhibitor cocktail (catalog number 78430, Thermo Fisher Scientific, Waltham, MA, USA) according to the weight of the tissue and centrifuged at 4 °C (10,000 × *g* for 20 min). The expression of cytokines in the mPFC and hippocampus was simultaneously measured by using a kit (Bio-Plex Mouse Cytokine Group I, 4-Plex Assay kit; Bio-Rad Laboratories, Hercules, CA, USA). A Luminex System kit was used to detect TNF-α, and its levels were quantified by using Bio-Plex 6.0 (Bio-Rad). Plasma TNF-α level was also measured by using an enzyme-linked immunosorbent assay (ELISA) kit (R&D, RTA00 for TNF-α). Cytokine concentrations in each brain sample were standardized to the buffer dilution with the tissue weight or the total protein concentration of the tissue.

### Detection and quantification of plasma and brain Aβ-42

The concentration of beta-amyloid 1–42 (Aβ-42) in the plasma and cortex was measured by using an ELISA kit (KMB3441, Invitrogen, Carlsbad, CA, USA). The cortex was homogenized according to the manufacturer’s instructions. Briefly, the cortex tissue was weighed and homogenized in eight volumes of cold solution A (5 M guanidine HCl in 50 mM Tris–HCl, pH 8.0). The homogenate was maintained at room temperature for 3 to 4 h and then diluted with solution B (0.2 g/L KCl, 0.2 g/L KH_2_PO_4_, 8 g/L NaCl, 1.150 g/L HPO_4_, 5% bovine serum albumin, and 0.03% Tween-20, pH 7.4), supplemented with 1X protease inhibitor cocktail (catalog number 78430, Thermo Fisher Scientific). The samples were then centrifuged at 16,000 × *g* for 20 min at 4 °C, and the supernatants were collected and kept on ice until Aβ quantification. The plasma and cortex supernatants were then used to evaluate the levels of Aβ-42 in the plasma and brain.

### Immunohistochemical analysis and immunofluorescence staining of brain tissue

The right hemispheric brain was dissected and placed in 4% paraformaldehyde in 0.1 M phosphate buffer overnight at 4 °C. After the brain had been suspended in a sucrose solution (10–30%), it was embedded in optimal cutting temperature compound and frozen immediately at − 80 °C. Serial transverse brain slices (30 μm) from the PFC and hippocampus were sectioned by using a cryostat. To facilitate the detection of microglia, we incubated the brain sections for 24 h at 4 °C in 5% goat serum mixed with PBS with 0.1% tween 20 (PBST) that contained anti-IbA1 (1:2000, WAKO-016–20,001). After the sections had been repeatedly washed in PBST, they were incubated in 1:1000 dilutions of biotinylated secondary antibody (BA-1000, Vector Laboratories, Peterborough, UK) and then in an avidin–biotin complex (Elite kit; Vector). The peroxidase reaction product was visualized by incubating the sections for 2 min in a solution that contained 0.022% 3,30-diaminobenzidine (DAB) (Vector).

For immunofluorescence staining, slices from each area of the brain were incubated at 4 °C in 5% bovine serum albumin (BSA) in PBST with antibody against astrocyte marker glial fibrillary acidic protein (GFAP, Abcam, ab7260, 1:1000), phosphorylated tau (Invitrogen, MN1040, 1:200), or neuronal cell marker NeuN (Merk Millipore, ABN78, 1:200) for 24 to 48 h at 4 °C. After washing with PBST, the sections were treated with secondary antibody against rabbit IgG coupled to Alexa Fluor 488 (Invitrogen, 1:100). DAPI (4′ ,6-diamidino-2-phenylindole) was used as a nuclear counterstain. Finally, the sections were rinsed and mounted with Shandon Immu-Mount (Thermo Scientific) or Micromount solution (M-3801730, Leica Camera AG, Wetzlar, Germany). The processed sections were analyzed for microglia or astroglia by using an upright microscope (BX53; Olympus Global, Tokyo, Japan), a camera (DP73; Olympus), and an authorized imaging software (cellSens Dimension software, version 1.3; Olympus).

### Western blot analysis

The hippocampus was isolated from mice, homogenized, and lysed in 1X cell lysis buffer (diluted from 10X cell lysis buffer, catalog number #9803, Cell Signaling Technology) supplemented with 1 mM phenylmethanesulfonyl fluoride (catalog number P7626, Sigma-Aldrich). The homogenate was centrifuged at 4 °C for 10 min at 10,000 × g. The supernatant was collected for protein analysis. Equal amounts of protein (30 μg) were loaded and separated by using 4% to 12% sodium dodecyl sulfate polyacrylamide gel electrophoresis (SurePAGE, Bis–Tris, GenScript, Singapore**)** and were then transferred to polyvinylidene difluoride (PVDF) membranes. The PVDF membranes were blocked with 5% BSA in Tris-buffered saline with 0.1% Tween 20 (TBST) for 1 h and then incubated with primary antibodies for VLDLR (Abcam, ab203271, 1:200), phospho-PI3K (Cell Signaling Technology, Danvers, MA, USA, #4228, 1:500), PI3K (Invitrogen, #PA5-29,220, 1:1000), or GADPH (Santa Cruz Biotechnology, Inc., Dallas, TX, USA, sc-32233, 1:2000) for 12 h at 4 ^◦^C. The membranes were then incubated with secondary antibodies for 1 h at 25 °C. All blots were incubated and visualized with enhanced chemiluminescence Western blot detection reagents (Thermo Fisher, 34,577).

### Statistical analysis

Data were analyzed by using one-way analysis of variance and then Newman-Keuls post-hoc test. Prism 5 and SPSS 22 were used to perform statistical analyses. Data are expressed as mean ± standard error of the mean. Significance was set at p < 0.05.

## Supplementary Information

Supplementary Information
