# Supplementary material for: Electronegative very-low-density lipoprotein induces brain inflammation and cognitive dysfunction in mice
Source: Sci Rep. 2021 Mar 16;11:6013. doi: 10.1038/s41598-021-85502-0 (PMC7966811; doi:10.1038/s41598-021-85502-0)
Supplement: Supplementary file 1 — Supplementary Information [file 41598_2021_85502_MOESM1_ESM.docx]

**Electronegative very-low-density lipoprotein induces brain inflammation and cognitive dysfunction in mice**

Ying-Shao Lin^1†^, Ching-Kuan Liu^1,2,3†^, Hsiang-Chun Lee^4,5^, Mei-Chuan Chou^3,6,7^, Liang-Yin Ke^5^, Chu-Huang Chen^1,5,8^, & Shiou-Lan Chen^1,9*^

^1^Graduate Institute of Medicine, College of Medicine, Kaohsiung Medical University (KMU), Kaohsiung, Taiwan. ^2^Department of Neurology, KMU Hospital, Kaohsiung, Taiwan. ^3^Department of Neurology, Faculty of Medicine, College of Medicine, KMU, Kaohsiung, Taiwan. ^4^Division of Cardiology, Department of Internal Medicine, KMU Hospital & Department of Internal Medicine, Faculty of Medicine, College of Medicine, KMU, Kaohsiung, Taiwan. ^5^Lipid Science and Aging Research Center, College of Medicine, KMU, Kaohsiung, Taiwan. ^6^Graduate Institute of Clinical Medicine, College of Medicine, KMU, Kaohsiung, Taiwan. ^7^Department of Neurology, Kaohsiung Municipal Ta-Tung Hospital, KMU, Kaohsiung, Taiwan. ^8^Vascular and Medicinal Research, Texas Heart Institute, Houston, Texas, USA; ^9^Department of Medical Research, KMU Hospital & MSc Program in Tropical Medicine, College of Medicine, KMU, Kaohsiung, Taiwan.

^†^These authors contributed equally to this work.

**^*^Corresponding author**

Shiou-Lan Chen, PhD (ORCID: 0000-0003-3995-0276)

Graduate Institute of Medicine, and Lipid Science and Aging Research Center, College of Medicine, Kaohsiung Medical University & Hospital, Kaohsiung, Taiwan

100 Shiquan 1st Rd, Sanmin Dist., Kaohsiung City 807, Taiwan.

Tel: +886-7-312-1101 ext. 5092, ext. 434; E-mail: shioulan@kmu.edu.tw.

Supplementary data


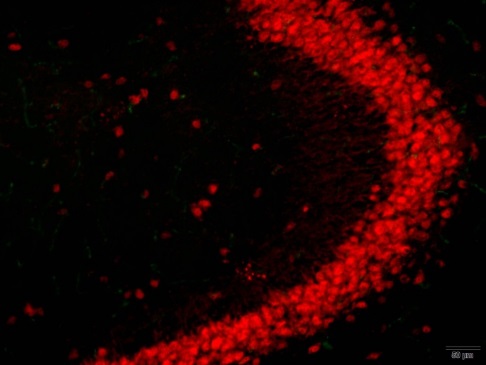

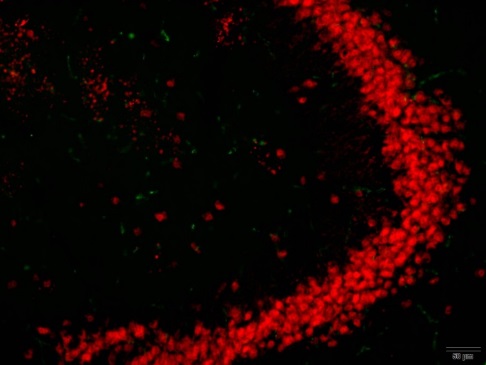

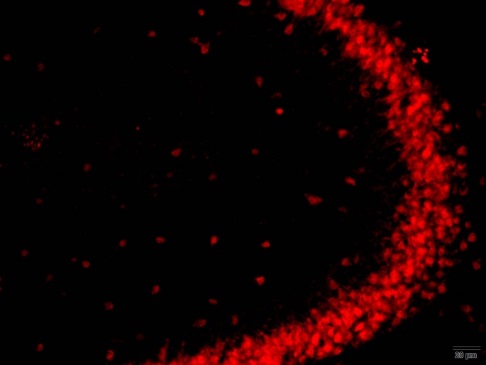


a. Control

b. nVLDL

c. metVLDL

NeuN: 100%

(TNF-α: 4.3 pg/mg protein)

NeuN: 63.2%

(TNF-α: 34.5 pg/mg protein)

NeuN: 80.8%

(TNF-α: 11.7 pg/mg protein)

Figure S1. Immunofluorescence staining showing the expression of NeuN (neuronal cells, red) in the hippocampus CA3 of mice after 6 weeks of injections with (a) saline (control), (b) 15 μg/g nVLDL, or (c) 15 μg/g metVLDL. Magnification = 20X; scale bar = 50 μm.

**GADPH (37 KDa)**

**1 2 3 1 2 3 1 2 3**

**p-PI3K (85 KDa)**


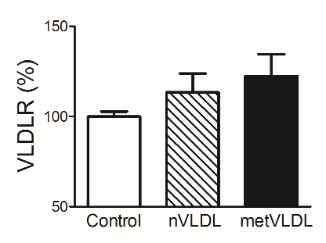

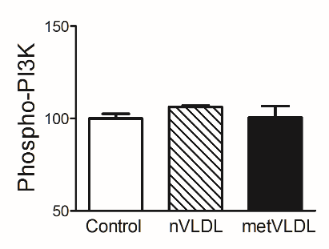


Hippocampus

**Control nVLDL metVLDL**

a

b

**PI3K (85 KDa)**


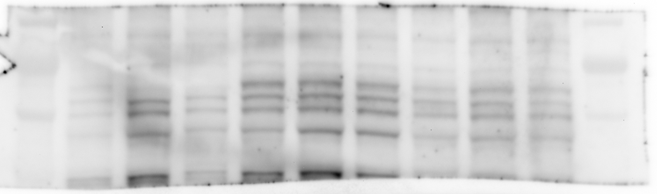

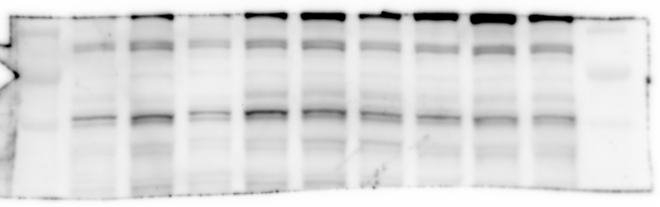

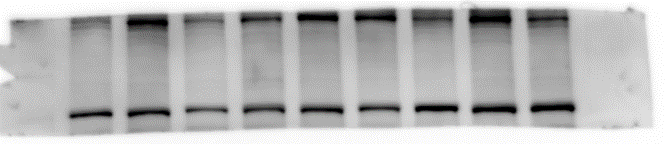


**100**

**35**

**KDa**

**180**

**130**

**75**

**63**

**48**

**100**

**75**

**63**

**48**

**VLDLR (130 KDa)**


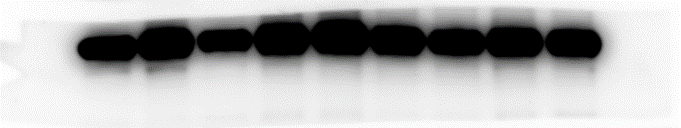


Figure S2. Expression of VLDL receptor (VLDLR), phospho-PI3 kinase (p-PI3K), and PI3K in the hippocampus of mice. (a) Immunoblotting results after 6 weeks of injections with saline (control), 15 μg/g nVLDL, or 15 μg/g metVLDL. (b) Percentage (%) of immunoblotting signals for VLDLR and phosphorylated PI3K (n ≥ 3). Data are presented as the mean ± SEM (one-way ANOVA).
